# Report on the Use of Pressure in the Treatment of Gonorrhœal and Purulent Ophthalmia

**Published:** 1865-11

**Authors:** Jos. S. Hildreth

**Affiliations:** U.S.V., in charge of Desmarres (U.S. Army) Eye and Ear Hospital, Chicago


### REPORT ON THE USE OF PRESSURE IN THE TREATMENT OF GONORRHŒAL AND PURULENT OPHTHALMIA.

By Surgeon JOS. S. HILDRETH, U.S.V.,
In charge of Desmarres (U.S. Army) Eye and Ear Hospital, Chicago.

Read before the American Ophthalmological Society, June 13th, 1865.

Tabular Statement *of all Cases of Gonorrhœal and Purulent Ophthalmia treated in the Desmarres Eye and Ear Hospital, at Chicago, from Aug.* 26, 1864, *to January* 15, 1865, *with results obtained.*

| No. | Names. | Age. | Affection. | Treatment by Pressure. With. Right. | With. Left. | Without. Right. | Without. Left. | Results. Saved. Right. | Saved. Left. | Lost. Right. | Lost. Left. |
|---|---|---|---|---|---|---|---|---|---|---|---|
| 1 | Chapin ..... | 23 | Purulent Ophthalmia... | 1 | 1 | ... | ... | 1 | 1 | ... | ... |
| 2 | Case, ........ | 48 | " " | 1 | 1 | ... | ... | 1 | 1 | ... | ... |
| 3 | Richards | 33 | " " | 1 | 1 | ... | ... | 1 | 1 | ... | ... |
| 4 | Haight ... | 26 | " " | ... | 1 | 1 | ... | ... | 1 | 1 | ... |
| 5 | Vosburgh | 43 | " " | 1 | ... | ... | 1 | 1 | ... | ... | 1 |
| 6 | Finn......... | 23 | " " | 1 | ... | ... | 1 | 1 | ... | ... | 1 |
| 7 | Shimming | 20 | " " | 1 | ... | ... | 1 | 1 | ... | ... | 1 |
| 8 | Ulrich...... | 25 | " " | 1 | ... | ... | 1 | 1 | ... | ... | 1 |
| 9 | Strong...... | 24 | " " | ... | ... | 1 | 1 | 1 | 1 | ... | ... |
| 10 | Shafer...... | 39 | " " | ... | ... | 1 | 1 | ... | 1 | 1 | ... |
| 11 | Smith ...... | 25 | " " | ... | ... | 1 | 1 | 1 | 1 | ... | ... |
| 12 | Doran ...... | 17 | " " | ... | ... | 1 | 1 | ... | ... | 1 | 1 |
| 13 | Gibbs ...... | 43 | " " | ... | ... | 1 | 1 | ... | ... | 1 | 1 |
| 14 | Ellsworth | 24 | Gonorrhœal Ophthalmia | 1 | 1 | ... | ... | 1 | ... | ... | 1 |
| 15 | Essen ...... | 40 | " " | 1 | 1 | ... | ... | 1 | 1 | ... | ... |
| 16 | Mervin ... | 20 | " " | ... | ... | 1 | 1 | 1 | 1 | ... | ... |

Recapitulation.

| | No. | Saved. | Lost. |
|---|---|---|---|
| Eyes Treated with Pressure ........................................ | 15 | 14 | 1 |
| Eyes Treated without Pressure ..................................... | 17 | 7 | 10 |

The condition of the above cases at the commencement of treatment with pressure was as follows. Results are also given.

CONDITION OF PATIENTS.

No. 1.—Corneæ panniform and ulcerated; chemosis sero-phlegmonous and large; purulent discharge abundant.

Nos. 2 and 3.—Corneal épithelium of both eyes considerably disturbed; chemosis sero-phlegmonous, firm and large; discharge abundant.

RESULTS, &c.

Pressure was used on both eyes in these three cases.

No. 1 recovered in good condition, except nebulous spots at seat of old ulcerations of corneæ, whose panniform condition is fast disappearing and vision improving.

Nos. 2 and 3 recovered in good condition.

CONDITION OF PATIENTS.

No. 4.—Right cornea sloughed and lost; left corneal epithelium so disturbed as to seriously interfere with its transparency; chemosis large and phlegmonous.

No. 5.—Cornea of left eye sloughed and lost; right corneal epithelium considerably disturbed; chemosis phlegmonous, large and firm.

No. 6.—In similar condition.

No. 7.—Left cornea sloughed and lost; right affected with central ulceration and perforation; chemosis phlegmonous and very large; great tumefaction of the lids of both eyes.

No. 8.—Left cornea sloughed and lost; right panniform and ulcerated; chemosis very large, firm an l phlegmonous; purulent discharge from all abundant.

RESULTS, &c.

Pressure was employed on left eye of No. 4 and on right eye of Nos. 5, 6, 7, and 8.

Nos. 4, 5, and 6 recovered in good condition; No. 7 with central leucomatous spot, artificial pupil practicable; and No. 8 with cornea cloudy and panniform, but constantly improving.

CONDITION OF PATIENTS.

Nos. 9, 10, and 11.—Severe purulent opthalmia, with large sero-phlegmonous chemosis, (No. 10 being phlegmonous;) purulent discharge abundant, and great tumefaction of lids.

RESULTS, &c.

No pressure was used in these cases.

No. 9 recovered both eyes in good condition. No. 10 lost left eye from sloughing of cornea, the right cornea recovering, panniform and nebulous, but improving. No. 11 recovered with synechia anterior of each eye, from perforation of the corneæ.

CONDITION OF PATIENTS.

Nos. 12 and 13.—Severe purulent opthalmia, accompanied by considerable disturbance of corneal epithelium of both eyes and large sero-phlegmonous chemosis; purulent discharge very abundant; lids greatly tumefied.

RESULTS, &C.

No pressure used, and both eyes lost in each case from sloughing of the corneæ.

CONDITION OF PATIENT.

No. 14.—Cornea of left eye largely infiltrated in its deep "laminæ;" very large and firm chemosis; lids largely tumefied and eye scarcely influenced by recti-muscles; discharge of gonorrhœal pus from both eyes very profuse. Cornea of right eye slightly infiltrated in superficial "laminæ;" chemosis large, phlegmonous and firm; lids much swollen; patient had discharge from the urethra.

RESULTS, &C.

Pressure employed on both eyes. Cornea left eye recovered, opaque from ulceration and perforation; cornea of right eye slightly cloudy, but vision continually improving.

CONDITION OF PATIENT.

No. 15,—Epithelium of both corneæ considerably disturbed; chemosis large, firm and phlegmonous, great tumefaction of lids, and abundant gonorrhœal discharge.

RESULTS, &C.

Pressure applied to both eyes, which recovered in good condition.

CONDITION OF PATIENT.

No. 16.—Corneal epithelium slightly disturbed; large phlegmonous chemosis; great tumefaction of lids, and abundant gonorrhœal discharge from both eyes.

RESULTS, &C.

No pressure used. Recovered in good condition.

From the preceding table and subsequent remarks, it appears that thirteen patients with purulent ophthalmia were treated. In three cases both eyes with pressure, and both eyes in each case recovered.

In five cases an eye only of each patient was treated with pressure, and the other eye without. All the former were saved, and all the latter lost.

In five cases not treated with pressure, one patient recovered both eyes in good condition; one with synechia anterior of each eye; one losing the right and saving the left eye, and two losing both eyes.

Three patients affected with gonorrhœal opthalmia were also treated.

In two cases both eyes were treated with pressure. One patient recovered both eyes, and one lost the left and saved the right eye.

In one case in which no pressure was used both eyes were saved.

The above comprises all cases of these types treated in the hospital from August 26th, 1864, to January 15th, 1865, which I have been particular to describe in order to show the nature of these affections as they manifested themselves, and the relative value of the application of pressure in their treatment.

Those of purulent ophthalmia exhibited an unusual degree of malignity; especially in tendency to destruction of the cornea by infiltration or ulceration, and sloughing "couche sur couche."

Some oculists have suggested, what occurred to me at the time, that many of them were diphtheritic, but I am not satisfied such was the case. At all events, the indications of such disease were as well marked in those treated with pressure as in the others. The cases of gonorrhœal ophthalmia presented nothing unusual beyond what has been described above.

I have been careful and explicit in regard to details, on account of the marked difference in the results obtained with and without pressure in the treatment, which, in all other respects, was the same; and the more so, as in five cases circumstances placed it in my power to witness the difference of treatment on the same individuals.

This became possible from the fact of my entire want of knowledge of the use of pressure in such cases, until circumstances forced me to devise some method of staying the ravages of disease over which I could obtain, by all known means, but little control; and it was not until ten eyes out of twenty-four had been lost, that the idea suggested itself to me, which, like many others, was at once carefully and prudently acted upon.

Twelve hours seemed to justify the means adopted, and after twenty-four hours the change in the left eye of case No. 4, the only one at first attempted, was so decisive as to warrant the experiment on a more extended scale.

The results are given in the table.

What I mean by the use of pressure in the treatment of such cases, is not the application of lint, wet or dry, over the lids with moderate compression, but a *firm, hard, continued pressure upon all parts of the contents of the orbit, especially the anterior.* This I effect in the following manner:—

The lids being closed, the orbit is to be packed, as it were, by means of charpie, or picked lint, (scraped lint or cotton wool is not so serviceable,) in such a manner that all parts about the eye, within the orbit, the anterior hemisphere of the globe, and especially the conjunctiva, shall be acted on.

Care must be taken to fill the grand angle, and to have the charpie evenly and regularly disposed *about* as well as over the globe.

Quite a large bunch should be used for each eye, not only to ensure evenness of pressure, but to absorb the purulent discharge. This being done, compression is made by means of a bandage, or better, a firm elastic band of rubber braid, not less than two inches in width, passing around the head. It should be slowly and regularly increased until the pain, if any there be, in the parts affected, is greatly diminished or controlled, if practicable.

In other words, pressure is to be applied to the eye and surrounding parts within the margin of the orbit to a decree sufficient to so control the circulation as to prevent the destructive tendency of the disease, but not to interfere with proper nutrition. This must, of course, vary with the peculiarities of each case.

But the principle of employing, as constantly as possible, firm, hard, even and continued pressure from the *earliest moment practicable* until the *close of all acute symptoms*, is not to be lost sight of for a moment. The anatomy of the orbit, the mechanism of the lids, and the cushion of adipose tissue posterior to the globe, render this not only possible, but easy.

I have in no instance resorted to it in purulent or gonorrhœal affections of the eye during the acute stages, even after the organ has been irretrievably lost, without greatly diminishing the discharge in a short time, and very materially adding to the patient's comfort in reducing the pain, and modifying subsequent and present staphyloma, as occurred in cases numbered 4, 5, 6, 7, 8, and 12.

While the purulent discharge is abundant, the dressing should be renewed twice during every twenty-four hours. Dry charpie is to be preferred, though moist will answer; yet it is not so elastic.

That pressure will have a potent influence in diminishing the discharge, reducing the tumefaction of the lids and the chemosis, modifying extravasation and exudation, arresting and inducing infiltration of the cornea to become resolved, is now a clinical fact.

The rationale of such action is certainly as simple as the means employed to produce it.

The influence of the virus (be it what it may) induces an extraordinary flow of blood to the affected parts, and often with great rapidity.

Their arterial circulation is taxed to the utmost, and the venous also; while the capillary connecting them is inadequate to the demand imposed upon it, even when distended to its utmost.

Hence the results which unhappily too frequently follow. Compression of the affected parts diminishes the flow of blood into them, so acts upon the capillaries as to prevent their enlargement, stimulating them to perform their functions, besides producing partial anæsthesia, and controlling or modifying the pain.

Having dwelt sufficiently, I think, upon the uses of pressure in these cases, I now propose to allude to the treatment I have found in other respects most beneficial. Before doing so, a division of cases will be desirable, to illustrate my reasons for adopting certain means.

1st. Those cases in which pain, swelling, heat, redness, and phlegmonous or phlegmono-serous chemosis are decided and well-marked, and which, usually occuring in patients of full habit, or having that condition favorable to the formation of the so-called "plastic lymph," may be called Sthenic.

2d. Those characterized by "serous puffiness" of the lids, serous or sero-phlegmonous, chemosis, little pain, discharge thin, and great tendency to infiltration on the part of the cornea—conditions which usually occur with persons whose systems have been reduced by scurvey, typhoidal disease, chronic diarrhœa, &c.—and which may be termed Asthenic.

Before proceding to detail the medical, I must allude to certain surgical means, frequently found necessary. It often occurs that the cornea becomes anæsthetized, so much so that the patient feels very imperceptibly the contact of a foreign body—as the point of a small roll of twisted paper, or a small camel's hair brush—and the pupil cannot be influenced by atropia, or only partially so, though *no* adhesions exist between the iris and the capsule.

I have found infiltration of the cornea to follow very closely upon such complications. Deep scarifications, circular or radiated, of the chemosis, or cups to the temple, have, in my hands, been very unsatisfactory in removing or preventing such conditions. No better results seem to follow paracentesis of the anterior chamber, "repeated" or otherwise. Unless largely infiltrated, I have frequently succeeded in saving the cornea in such cases by means of Hancock's operation of division of the "ciliary ring." Besides its preventing infiltration and sloughing of that important membrane, the patient will suffer much less pain during the continuance of acute symptoms. Unless the cornea is in an anæsthetized condition, is beginning to be infiltrated, or shows symptoms of sloughing and ulcerating, such an operation should not be resorted to.

I have frequently found the indications for this operation, as mentioned above, to exist with other affections of the eye, and have relieved them in the same manner; but the subject would, of itself, form an extensive article, and I shall therefore not dwell longer upon it here, beyond remarking that Hancock's operation in relieving such symptoms and conditions, cannot be relied upon to take the place of pressure; neither will the latter, under similar circumstances, relieve the necessity of dividing the "ciliary ring."

The utility of scarifications, deep, circular or radiated, of the chemosis is too well known to be dwelt upon here.

For local application I rely mainly upon bromide of ammonium, atropia, and nitrate of silver. In sthenic cases I prefer the use of bromide of ammonium dissolved in glycerine—forty to sixty grains to an ounce of pure glycerine—which is applied twice daily to the conjunctiva, ocular and palpebral, by means of a camel's hair brush.* It may be employed oftener in some cases, but this will be found, as a general rule, sufficient. Under its influence purulent, and especially gonorrhœal ophthalmia, appears to become rapidly modified, as I have frequently had occasion to demonstrate. The addition of ten grains of tannin to one ounce of the solution adds somewhat to its efficacy, but this is not indispensible.

For asthenic cases the nitrate of silver is most serviceable. I prefer to apply it gently to the mucous membrane of the lids,

* The following will be found serviceable for gonorrhœa:—

| | |
|---|---|
| Bromide of ammonium, | dr. ss.—dr. j. |
| Tannin, | dr. ij |
| Aqua, | oz. ij.—Misce. |

*Sig.*—One half ounce to be injected *pro re nata.*

neutralizing any excess of the salt by proper means. Blood may or not be taken from the lids, the chemosis or the temple, after the use of bromide of ammonium or nitrate of silver; but this must depend on the size of the chemosis and state of the patient. Atropia will be required to dilitate and so maintain the pupils.

For general treatment in sthenic cases I prefer muriate of ammonia in alterative doses, from three to five grains every one or two hours. Asthenic cases are benefitted by muriated tincture of iron, five drops every two hours or oftener, if the patient will bear it. Permanganate of potassa is also useful, in ¼ grain doses, every two or three hours. But it is evident that all general means must be adapted to the existing condition of the patient. The treatment for purulent and gonorrhœal ophthalmia may, therefore, be summed up as follows:—

1st. If anæsthesia of the cornea exists, or it is infiltrating, and especially if the pupil will not yield to the influence of atropia, Hancock's operation of division of the "ciliary ring" is indicated, care being taken to divide all its fibres from the insertion of the iris to its posterior limit.

2d. Application of a solution of bromide of ammonium, (40 to 60 grs. to ℥j. pure glycerine,*) or nitrate of silver to conjunctiva; the former to all parts of the conjunctiva, and the latter to that covering the cartilage of the lids only.

3d. Scarification of the lids and deep incisions into the chemosis, if required, removing the blood with tepid water so long as it continues to flow.

4th. Atropia in solution (iv. grs.–℥j.) sufficient to dilate the pupil.

5th. Application of firm, hard, continued pressure, as soon as practicable, and continued to the close of acute symptoms.

6th. Remove the dressings twice during every twenty-four hours, until the purulent discharge ceases.

7th. Two applications daily of bromide of ammonium or one of nitrate of silver will be found sufficient. Atropia may be used twice daily or oftener, but care should be taken not to continue its employ beyond *producing* and *maintaining moderate* dilatation of the pupil.

8th. A constitutional treatment adapted to the condition of the patient.

It is evident that no single remedy or means should be exclusively relied on in the treatment of purulent or gonorrhœal ophthalmia; but each case, and even each eye, must be managed

* Glycerine perfectly pure should be used.

in accordance with its existing conditions, and the varying symptoms promptly met by appropriate means. In this way we shall be justified in prognosticating favorable results in most cases.

In closing, it is sincerely hoped the special principle of treatment so prominently set forth in this article, as well as all others having anything unusual of application or otherwise will be rigidly tested, and the results made known to the profession.

